# Extracellular Vesicles and Pancreatic Cancer: Insights on the Roles of miRNA, lncRNA, and Protein Cargos in Cancer Progression

**DOI:** 10.3390/cells10061361

**Published:** 2021-06-01

**Authors:** Roberta Romano, Anna Picca, Leonardo Henry Umberto Eusebi, Emanuele Marzetti, Riccardo Calvani, Loredana Moro, Cecilia Bucci, Flora Guerra

**Affiliations:** 1 Department of Biological and Environmental Sciences and Technologies, University of Salento, 73100 Lecce, Italy; roberta.romano@unisalento.it; 2 Fondazione Policlinico Universitario “Agostino Gemelli” IRCCS, 00168 Rome, Italy; anna.picca1@gmail.com (A.P.); emanuele.marzetti@policlinicogemelli.it (E.M.); riccardo.calvani@gmail.com (R.C.); 3 Aging Research Center, Department of Neurobiology, Care Sciences and Society, Karolinska Institute and Stockholm University, 171 77 Stockholm, Sweden; 4 Gastroenterology and Endoscopy Unit, IRCCS Azienda Ospedaliero-Universitaria di Bologna, 40138 Bologna, Italy; leonardo.eusebi@unibo.it; 5 Department of Medical and Surgical Sciences (DIMEC), University of Bologna, Gastroenterology and Endoscopy Unit, Sant’Orsola University Hospital, 40138 Bologna, Italy; 6 Institute of Internal Medicine and Geriatrics, Università Cattolica del Sacro Cuore, 00168 Rome, Italy; 7 Department of Biochemistry and Molecular Pharmacology, New York University Grossman School of Medicine, New York, NY 10016, USA; Loredana.Moro@nyulangone.org or; 8 Perlmutter NYU Cancer Center, New York University Grossman School of Medicine, New York, NY 10016, USA; 9 Institute of Biomembranes, Bioenergetics and Molecular Biotechnologies, National Research Council, 70126 Bari, Italy

**Keywords:** pancreatic cancer, exosomes, extracellular vesicles, miRNA, lncRNA, inflammation

## Abstract

Pancreatic cancer (PC) is among the most devastating digestive tract cancers worldwide. This cancer is characterized by poor diagnostic detection, lack of therapy, and difficulty in predicting tumorigenesis progression. Although mutations of key oncogenes and oncosuppressor involved in tumor growth and in immunosurveillance escape are known, the underlying mechanisms that orchestrate PC initiation and progression are poorly understood or still under debate. In recent years, the attention of many researchers has been concentrated on the role of extracellular vesicles and of a particular subset of extracellular vesicles, known as exosomes. Literature data report that these nanovesicles are able to deliver their cargos to recipient cells playing key roles in the pathogenesis and progression of many pancreatic precancerous conditions. In this review, we have summarized and discussed principal cargos of extracellular vesicles characterized in PC, such as miRNAs, lncRNAs, and several proteins, to offer a systematic overview of their function in PC progression. The study of extracellular vesicles is allowing to understand that investigation of their secretion and analysis of their content might represent a new and potential diagnostic and prognostic tools for PC.

## 1. Introduction

Pancreatic cancer (PC), in all forms, is among the most common and devastating digestive tract cancers worldwide. The majority of PC cases (over 85%) are attributed to pancreatic ductal adenocarcinoma (PDAC) [1]. Due to the frequently late diagnosis, the suboptimal efficacy of available therapies, and the unpredictable progression, the five-year overall survival rate of PC is smaller than 5% and the life expectancy of advanced PC is only 3–6 months [2,3]. 

Surgical resection is the only potential curative treatment, but this option is possible in only 10–15% of patients [4]. Novel chemotherapeutic regimens have recently been devised and are based on the administration of FOLFIRINOX (a combination of 5-fluorouracil/leucovorin with irinotecan and oxaliplatin) and gemcitabine plus nab-paclitaxel; yet, their overall efficacy remains limited [5,6,7,8]. 

PDAC is characterized by the accumulation of mutations in key oncogenes and oncosuppressor genes that ultimately induce tumor growth and evasion from the immune system [9]. Pancreatic intraepithelial neoplasia (PanIN), intraductal papillary mucinous neoplasm (IPMN), and mucinous cystic neoplasm (MCN) are recognized as PDAC precursor lesions [10]. Pancreatic tumorigenesis results from the dysregulation of several core pathways and a myriad of genomic alterations [11]. Human pancreatic cancers have a specific mutational signature that include near-ubiquitous activating mutations of the oncogene *KRAS* (K Rat Sarcoma virus) and the frequent inactivation of the tumor suppressors *TP53* (Tumor Protein P53), *SMAD4/DPC4* (SMAD family Member4/Deleted in Pacreatic Carcinoma 4) e *CDKN2A* (Cyclin-Dependent KiNase inhibitor 2A) [12]. In general, *KRAS* mutations occur in stage 1 lesions (PanIN-1) and favor the initiation process; in PanIN-2, *CDKN2A* mutations emerge to induce further progression, while in stage 3 lesions (PanIN-3) and in invasive tumors, mutations of *TP53* and *SMAD4* drive the proliferation and expansion of cancer [12,13]. Unfortunately, the underlying mechanisms that orchestrate PC initiation and progression are poorly understood. Moreover, local and systemic inflammation has been identified as key contributor to the promotion of tumor growth and dissemination of PC [14]. The infiltration of inflammatory cells within the tumor microenvironment (TME) is an alleged mechanism through which inflammation is linked to PC carcinogenesis [15]. Within the PC TME, myeloid-derived suppressor cells (MDSC), M2 polarized macrophages, and regulatory T-lymphocytes (T_reg_) are more abundant than M1 macrophages, dendritic cells (DCs), and effector CD4^+^ and CD8^+^ T lymphocytes [16]. In this immune setting the TME acquires an immunosuppressive phenotype and cancer thrives. Along with this, the installment of an inflammatory milieu may nourish cancer cells and establish a dynamic and versatile metabolic symbiotic TME [15]. Hence, the release of inflammatory mediators may support crosstalk between immune and cancer cells and soluble inflammatory cytokines are main messengers in this communication [14].

Exosomes are a subset of extracellular vesicles (EVs) characterized by endosomal origin which are produced in TME by stromal and transformed cells [17]. Body fluids, such as blood, saliva, pancreatic duct fluid, cerebrospinal fluid, and amniotic fluid, transport exosomes to distal tissues or organs where they act via autocrine and paracrine routes [18]. The activation of various signaling pathways in target cells may be modulated by EVs, thus playing key roles in the pathogenesis and progression of several pancreatic precancerous conditions [19,20]. Indeed, these EVs are implicated in the transformation of various precancerous lesions into PC and in the progression of PC toward more invasive and metastasizing forms through the activation of mechanisms of angiogenesis, cell migration, epithelial–mesenchymal transition (EMT), and apoptotic resistance [21]. EVs may also serve as cytokine/immune-suppressive mediator shuttles in this setting. In fact, EV-enriched cytokines/immune-suppressive mediators may target their release where it is mostly needed to elude the immune system and hijack the host inflammatory response [22]. This, in turn, creates an environment that fosters tumor growth and progression. Among the inflammatory molecules identified in this immunomodulatory scenario, pro-inflammatory interleukin (IL) 1, IL6, tumor necrosis factor alpha (TNF-α), anti-inflammatory IL10, and transforming growth factor beta (TGF-β) have been indicated as major factors [14]. 

In this review, we discuss the role of EVs in PC progression, diagnosis, and prognosis. Recent reviews have discussed in detail the role of EVs in PC [23,24,25]. Here, we focused on cargo molecules of EVs, such as micro RNAs (miRNAs), long non-coding RNAs (lncRNAs), proteins, and inflammatory cytokines, to highlight the relevance of EVs in the diagnosis and prognostication of PC.

## 2. Exosomes

Exosomes are EVs of endosomal origin with a diameter of 50–150 nm and with a spherical, disc, or cup shape [17]. This kind of EVs originates by the release of intraluminal vesicles (ILVs) after fusion of multivesicular bodies (MVBs) with plasma membrane [26,27]. MVBs are endosomal organelles that undergo maturation in late endosomes moving from the cell’s periphery to the center along microtubules. According to this model, MVBs are identified as newborn late endosomes derived from the maturation of early endosomes. Still, an alternative model considers MVBs intermediate transporters between early and late endosomes [28]. Beside these mechanistic definitions, MVBs may have two different fates. In particular, these organelles can fuse with other MVBs or with late endosomes, undergoing progressive acidification to maturate into lysosomes for cargo degradation, or can move toward the plasma membrane to fuse and release ILVs that, in extracellular space, become exosomes [26,27]. Indeed, ILVs have a small diameter (50–150 nm) and are identified as exosome precursors. Thus, exosome biogenesis is closely connected to that of MVBs. MVB biogenesis is driven by two separate mechanisms linked to either the endosomal sorting complex required for transport (ESCRT) or lipid rafts [27,29]. 

The ESCRT system consists of five cytosolic complexes (i.e., ESCRT 0, I, II, III, and vacuolar protein sorting (VPS) [30]), and the purification of exosomes from different cell culture types or biological fluids has proven the role of the ESCRT system in nanovesicle biogenesis. For this reason, ESCRT proteins are now used as exosomal markers [17]. 

Exosomes originate from different types of cells, but they all share similar structural proteins such as major histocompatibility complex class I and class II molecules (MHC I/II), annexins, ALG-2 interacting protein X (ALIX), tumor susceptibility gene 101 protein (TSG101), flotillin (FLOT1), integrins, and tetraspanins [31]. Tetraspanins belong to 4-transmembrane protein family, which are ~100-fold more enriched in exosomes than in their parental cells [32]. In particular, the three tetraspanins CD9, CD81, and C63 are acknowledged as exosomal markers for their abundance in exosomes [33]. These proteins can form homodimers or heterocomplexes with other proteins and can couple with cholesterol and gangliosides to generate TEM domains (tetraspanin-enriched domain). These domains enriched of tetraspanins can recruit integrins, proteases, and other related signal molecules thus generating specific platforms for signal transduction [34]. 

The MVB/exosome ESCRT-independent biogenesis can still ensue via specific membrane lipid composition. In line with this, it is known that endosomes have domains enriched in cholesterol and sphingolipids, named lipid rafts, which are able to curve inward and determine the formation of MVBs with the support of the pH gradient across the membrane [35]. Indeed, phospholipases regulate the synthesis of ceramides from sphingolipids and cone-shaped structures of ceramides, alone or associated with cholesterol, generating areas suitable for membrane deformation, and ILV budding without ESCRT assistance. The neutral sphingomyelinases (SMases), localized in the Golgi and also in the plasma membrane, catalyzes the conversion of sphingomyelin in ceramide for exosomal biogenesis. Notably, the inhibition of SMases resulted in reduction of exosome secretion in specific cell types [36]. For these reasons, the exosomal surface contains a variety of lipid raft microdomains such as caveolae that integrate caveolins as structural proteins with the property to resist against detergents. Exosomal lipids comprise cholesterol, ganglioside, sphingomyelin, (hexosyl) ceramide, phosphotidylserine, and phosphotidylethanolamine [37]. The mechanisms through which MVBs move towards the plasma membrane for exosomal release instead of fusing with lysosomes are presently unclear. Nevertheless, SNARE (soluble N- ethylmaleimide-sensitive fusion protein attachment protein receptors) proteins and small GTPases are involved in the interaction between specific proteins and lipids determining fusion of MVBs with the plasma membrane and exosome secretion [38]. Indeed, the upregulation of SNARE VAMP7 (vesicle-associated membrane protein 7) induces enlargement of MVBs, their clustering at the cell’s periphery, and inhibition of exosome release [39], while RAB proteins—such as RAB27, RAB35, and RAB7—seem to be essential for docking MVBs at the plasma membrane and for exosome secretion in the extracellular space [40,41,42,43].

After their release into the extracellular space, exosomes are delivered via body fluids to recipient cells. Upon interaction with target cell receptors, exosomes can activate specific signaling pathways through the recognition and conjugation with membrane-bound receptors. Alternatively, these nanovesicles can be internalized into recipient cells by different mechanisms, including clathrin-dependent endocytosis, lipid raft (caveolae/caveolin-1)-mediated endocytosis, and macropinocytosis or phagocytosis [44]. Once into the cells, exosomes follow three different routes: (1) they can fuse to MVBs and/or mature late endosomes being then transported to lysosomes in order to undergo degradation for exosomal component recycling; (2) they can be released into the cytoplasm to act as second messengers; (3) they can be secreted again as part of the transcellular transport [44].

The study of exosomes is generally not easy and, as referred by the International Society of Extracellular Vesicles (ISEV), the assignment of a particular biogenesis pathway to EVs remains very difficult to establish as it could be demonstrated only through live imaging assay of EV release [17]. Thus, due to current technical limitations, the great majority of studies are unable to isolate and study a pure population of exosomes. For this reason, in this review, we have used ‘EVs’ as a generic preferred term, which includes also exosomes, as indicated by ISEV.

EVs are characterized by high stability in extracellular fluids and circulation, and contain a plethora of bioactive molecules which are transferred locally and systemically [45,46]. Notably, the EV content might depend on the EV function and on the type of EV-producing cells. Typically, the EV cargo includes proteins, nucleic acids, lipid molecules, and inorganic components such as Ca^2+^ [47,48].

## 3. Extracellular Vesicles in Pancreatic Cancer

The role of EVs in mediating intercellular communication in cancer is widely recognized, with particular involvement in carcinogenesis, chemoresistance, and immunosuppression [49]. 

**Figure 1:**
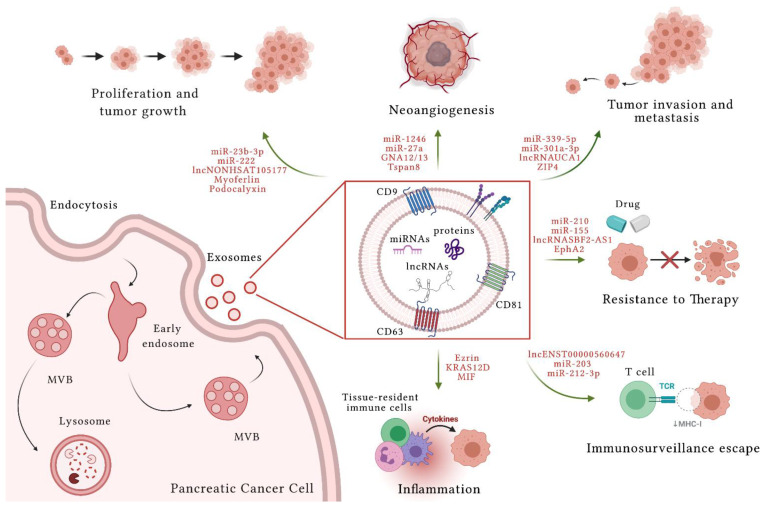
Role of extracellular vesicles (EV) in pancreatic cancer. Exosomes are subset of EVs. They originate after the fusion of multivesicular bodies (MVBs) with plasma membrane and the release of ILVs (Intraluminal vesicles) into extracellular space. The content of EVs determines their functions. EVs originated from pancreatic cancer (PC) cells deliver different functional biomolecules, such as micro RNAs (miRNAs), long noncoding RNAs (lncRNAs), and several proteins. In the figure examples of EV content are indicated. EVs play a pivotal role in PC progression. In fact, they are involved in proliferation and tumor growth, in the mechanisms of angiogenesis, in invasion and metastasis processes, in the induction of chemoresistance, in the escape of cancer immunosuppression, and in inflammation that creates a conducive microenvironment to cancer progression. Created with BioRender.com accessed on 20 May 2021.

In PC, EVs produced by cancer and non-cancer cells have a crucial role in TME communication and modulation of metabolic activity within TME and at distant sites. These nanovesicles are also involved in PC progression, invasion and dissemination, escape from immunosurveillance, and induction of chemoresistance [25] (Figure 1). Their role in both local and systemic cell–cell communication is exerted through the transfer of functional biomolecules, including miRNAs, other non-coding RNAs, and proteins, to recipient cells.

### 3.1. Pancreatic Cancer and Extracellular Vesicle miRNAs

Noncoding RNA (ncRNA) genes are essential constituents of the human genome, playing an important regulatory role. It is estimated that only one-fifth of all human genome transcripts encodes proteins, whereas the remaining is made of ncRNA genes [50]. Generally, ncRNAs can be classified on the basis of their size in small ncRNAs and lncRNAs. Micro RNA (miRNAs) and small interfering RNA (siRNA) are considered small ncRNA since they contain less than 200 nucleotides, whereas lncRNAs comprise a wide range of RNAs such as large intergenic ncRNAs (lincRNAs) and circular RNAs (circRNAs). MiRNAs (19–22 nucleotides) regulate post-transcriptional gene expression of their target mRNAs generally by binding to complementary seed sequences at the untranslated regions (3′-UTRs) [51]. Interactions of miRNAs with 5′-UTRs, coding sequences and gene promoters have also been reported [52]. Profiling of RNAs in the circulation has shown that miRNAs are involved in many pathological cancer processes [53,54]. Valadi et al. described for the first time miRNAs of EVs demonstrating that mast cells transport miRNAs, mRNAs, and other molecules to target cells [55]. Reports have identified EV miRNAs as novel bloodborne biomarkers for the early diagnosis of PC in light of their roles in biological processes such as cell proliferation, apoptosis, and differentiation [56,57]. Unlike circulating miRNAs, a key characteristic of EVs is the possibility to shield their cargo, in particular RNA, from RNase degradation abundantly presents in body fluids and in the TME [58]. The expression profile of intracellular miRNAs is different from miRNAs in EVs as a result of strict sorting mechanisms [59]. 

In general, miRNAs may play a dual role in carcinogenesis on the basis of the role of the target mRNAs in the tumor initiation process. Indeed, it is possible to distinguish oncosuppressor miRNA, when its downregulation increases the activity of a corresponding oncogene, and oncogenic miRNA (also termed oncomiRs), when its upregulation results in a persistent inhibition of the target tumor suppressor gene. Thus, alterations in the regulation of specific miRNAs determine their contribution to cancer development [60,61].

Accordingly, it has been demonstrated that pancreatic cancer tissue from PC patients and also PC cell lines overexpress miR-27a [62]. Interestingly, PC-derived EV delivering miR-27a can induce proliferation, invasion, and angiogenesis in human microvascular endothelial cells (HMVECs) through the suppression of B-cell translocation gene 2 (BTG2), which promotes PC cell survival and growth [63]. 

In contrast, miR-339-5p, which downregulates the zinc finger protein ZNF689, inhibits cell invasion and migration in PC animal models. In line with this, EV miR-339-5p levels are reduced in metastatic PC cells and the exogenous introduction of miR-339-5p reduces PC cell migration and invasion [63]. 

Wang and coauthors discovered that highly invasive PC cells overexpress miR-222 which is included into EVs. After uptake by poorly invasive PC cells, EV miR-222 is released to further induce downregulation, phosphorylation, and nuclear exit of p27 via the PPP2R2A (protein phosphatase 2 regulatory subunit B alpha)/Akt axis, which ultimately promotes cancer cell proliferation and invasion [64].

In general, it is known that hypoxia stimulates generation of small-volume EVs via HIF1α (Hypoxia Inducibile Factor 1 alpha), which increases the survival, proliferation, and dissemination of PC cells [65]. The production of EVs delivering miR-301a-3p in PC cells can be stimulated by hypoxic environment inside the tumor [66]. Upon uptake by other PC cells, miR-301a-3p promotes their metastatic ability and invasiveness. Furthermore, when macrophages are the recipient cells of this miRNA, they undergo HIF1α/2α-dependent M2 phenotype transformation due to activation of the PTEN (Phosphatase and Tensin Homolog)/PI3K (Phosphatidylinositol 3 Kinase) signaling cascade [66].

Patients with breast and prostate cancers showed abundance of circulating EV miR-1246 [67,68]. High levels of this miRNA are correlated with resistance to gemcitabine treatment in PC cells promoting PC metastasis, invasion, cancer stemness, and angiogenesis via inhibition of CCNG2 (clinical significance of cyclin G2) expression [69].

Additionally, chemosensitivity to gemcitabine was reduced in PC cells grown in conditioned media containing the EV fraction from gemcitabine-treated PC cells (Gem-Exo) [70]. Upregulation of reactive oxygen species (ROS) detoxification enzymes, such as catalase (CAT) and superoxide dismutase 2 (SOD2), and downregulation of deoxycytidine kinase DCK (gemcitabine-metabolizing gene) were described in Gem-Exo-treated cells [70]. Furthermore, EV miR-155 induces DCK downregulation and miR-155 suppression or DCK restoration, thereby abrogating Gem-Exo-mediated PC chemoresistance [70]. Accordingly, increased miR-155 expression was observed after long-term exposure of PC cells to gemcitabine. Indeed, this miRNA is able to control EV synthesis and secretion and to promote chemoresistance through antiapoptotic effects [71]. The targeting of miR-155 or EV secretion attenuated tumor chemoresistance, therefore representing a novel therapeutic approach to overcome gemcitabine resistance in PC [71]. 

Recently, it was also reported that gemcitabine-resistant PC stem cells secrete EVs which are able to enhance drug resistance in gemcitabine-sensitive PC cells by delivering miR-210. Indeed, miR-210 inhibits the effects of gemcitabine, such as cell cycle arrest and apoptosis, and promotes tube formation and cell migration leading to an EV-mediated increase of the invasive and metastatic potential of gemcitabine-treated cells [72]. 

Suppression of the miR-200 family in PC induces maintenance of stemness and promotes cancer cell migration by upregulating Zinc finger E-box-binding homeobox (ZEB)1 and ZEB2, which are important EMT mediators [73]. Recent evidence suggests that proliferation, migration and invasion are also promoted by EV-mediate transfer of miR-23b-3p, the production of which is increased in the PANC1 invasive PC cell line [74]. 

Regarding resistance to therapy, it is known that failure of radiotherapy can be due to cancer repopulation induced by radiotherapy-induced dying cancer cells (RI-DCCs). Indeed, it has been recently demonstrated that miR-196b-5p is enriched in EVs from pancreatic RI-DCCs, implying that it may serve as an EV-originating survival factor for irradiated cell. In this study, it was also shown that RI-DCCs secrete EVs containing miR-194-5p and transfer them to other cells. EV miR-194-5p is able to activate the DNA damage response, enhancing cell survival of receiving cells after radiation due to its ability to regulate target genes, such as E2F transcription factor 3 (E2F3) and high mobility group AT-hook 2 (HMGA2) [75]. 

### 3.2. Pancreatic Cancer and Extracellular Vesicle lncRNAs

As described above, lncRNA are endogenous RNA more than 200 nucleotide-long and with no protein-coding function. Albeit lncRNAs are still poorly understood relative to other non-coding transcripts, it is known that they are useful for transcripttional regulation. LncRNAs have specific localization in different cellular compartments which correlates with their different functions [76]. LncRNAs use a “decoy” or “sponge” mechanism to block DNA or RNA transcription and translation [77]. Indeed, they function as decoy to transcriptional regulators and as sponge to determine molecular scaffold, signaling regulation, or guide. Transcription factors are bound by lncRNAs that inactivate their function impeding them to reach their target. LncRNA may also act as molecular scaffold modulating gene expression by gathering proteins together, with the aim of forming functional ribonucleoprotein complexes that in turn activate o repress transcription. Acting as signaling molecules, lncRNAs send transcription proteins to particular genes to modify chromatin structure and promote transcription. When lncRNAs act as guide, they modulate gene activity and induce epigenetic changes through modification of chromatin architecture. All these functions can be achieved through lncRNAs interplay and translocation of selected enzymes, such as histone acetylases, deacetylases, and methylases, toward gene locations to alter chromatin ultrastructure [76].

In general, a dysregulation of lncRNAs has been described in several types of cancers and, in particular, abnormal regulation of miRNAs and lncRNAs holds a pivotal role in contributing to various hallmarks in PC [77,78,79]. In line with this, it was demonstrated that lncRNAs interacting with DNA, RNA and proteins are involved in PC pathogenesis influencing the tumorigenesis pathway at transcriptional, post transcriptional, and epigenetic levels [80].

Similar to miRNAs, lncRNAs are sorted into EVs and are crucial regulators for the pathogenesis and progression of PC and thus are considered promising biomarkers for this cancer type [80]. In fact, the X-linked inhibitor of apoptosis protein (XIAP) is a factor correlated with inhibition of PC progression through action of EV lncRNA. Indeed, XIAP inhibits apoptosis via caspases and its expression is repressed by miR-122-5p. Interestingly, the lncRNA SBF2-AS1 is overexpressed in EVs of PANC-1 cell line. This EV-delivered lncRNA increases the levels of miR-122-5p which in turn reduces XIAP expression suppressing PC progression [81]. Moreover, pancreatic cells cultured with PDAC-derived EVs delivering lncRNA NONHSAT105177 show decreased invasion and migration both in vivo and in vitro through modulation of the cholesterol biosynthesis pathway [82]. Another study suggested that EVs lncRNA could regulate intra- and extravasation of tumor cells demonstrating that angiogenesis was promoted by enrichment of lncRNA derived from immune-cell EVs. Moreover, in an hypoxic environment, EV lncRNA UCA1 is able to promote cell migration and tubulogenesis of endothelial cells. In particular, Guo et al. [83] have identified a mechanism of UCA1 action as sponge of miR-96-5p. Also, the increase of EV lncRNA SOX2OT cargo was recently associated with PC stages. Indeed, lncRNA SOX2OT can modulate EMT and its overexpression promotes invasion and metastasis in PC in vitro and in vivo [84]. 

A recent study has highlighted the importance of lncRNA also in the release of EVs in PC. In fact, lncRNA PVT1 is able to induce increase of EV secretion in PC cells by acting on YKT6 v-SNARE homolog (YKT6), Ras-related in brain 7 protein (RAB7), and vesicle-associated membrane protein 3 (VAMP3) [85]. 

### 3.3. Pancreatic Cancer and Extracellular Vesicle Proteins 

Besides miRNAs and lncRNAs, several studies indicate that EVs also contain numerous proteins with multiple roles in the progression of PC. In fact, small EVs (sEVs) released by PC cells may act as an initiator of malignant transformation, ability that sEVs secreted by normal pancreatic cells do not possess [86]. In addition, some proteins are uniquely included into sEVs secreted from PC cells (Capan-2, MIA PaCa-2, and Panc-1), others are uniquely present in sEVs of normal pancreatic cells, and other proteins are more abundant in vesicles derived from cancer cells compared to the normal counterpart [87]. To the first category belong Guanine Nucleotide-Binding Protein Alpha-12 (GNA12) and GNA13 [87]. They can stimulate cell growth through activation of Ras, Rac, Rho, CDC42 and their downstream pathways [88,89,90], which have been found to be hyperactivated in several cancers, contributing to tumor growth, angiogenesis, EMT, migration, and invasion [91]. Instead, protein members of RNA polymerase III with a role in immune response, such as RNA Polymerase 3 subunit B (POLR3B), POLR3A, POLR3D, and POLR3E have been found only in sEVs of normal pancreatic cells [87,92]. Among proteins more present in sEVs released by PC cells, RAB and ESCRT have been found [87] and their greater presence in sEVs of PC cells could be explained considering that cancer cells release more EVs than normal cells [30,93,94].

Some proteins overexpressed in various types of cancer have been found in EVs secreted by PC cells and are associated with enhanced ability of migration, invasion, and with drug resistance of the recipient cells [23]. A protein abundant in EVs of PC cells is the zinc transporter Zrt- and Irt-like protein 4 (ZIP4). In particular, high malignant PC cells released ZIP4-enriched EVs. These vesicles were able to increase proliferation, migration, invasion, abilities of moderate malignant recipient cells in vitro and promote cancer growth in vivo [95]. Also, asparaginyl endopeptidase (AEP), delivered by EVs secreted by PC cells, was shown to promote invasion. Indeed, this protein is able to activate the PI3K pathway in recipient cells leading to increased invasion in vitro [96]. Another protein abundant in PC EVs is the CD44 variant isoform 6 (CD44v6). In particular, it is included in EVs secreted by cancer-initiating cells (CICs) and stimulates migration and invasion through overexpression of plasminogen activator inhibitor 1 (PAI-1), metalloprotease (MMP), and tissue inhibitor of metalloproteases 1 (TIMP-1), but also promotes anchorage-independent growth and apoptosis resistance of PC recipient cells [97].

Moreover, a study analyzed the ability of EVs derived from PANC-1 and MIA PaCa-2 cells to promote metastasis by recruiting PSCs (pancreatic stellate cells). The authors showed that the cargo of these nanovesicles contained Lin-28 homolog B (Lin28B) that causes formation of metastases through the recruitment of PSCs [98,99].

Notably, it was shown that rat aortic epithelial cells acquired EVs derived from PC cells and that some proteins included in them, in particular Tetraspanins 8 (Tspan8), CD106 and CD49d, stimulate neovascularization, a process essential for angiogenesis and tumor growth [100]. The ability of EVs from PC to generate metastasis is reduced by TSPAN8 or CD151 knockout, demonstrating that both these proteins are essential for the formation of a pre-metastatic niche [101].

Another protein delivered by EVs of PC cells is myoferlin. The role of this protein in stimulating migration and invasion of pancreatic cancer cells has been linked to its function in the regulation of mitochondrial organization and energy production [102,103]. In PC cells, on one hand, myoferlin stimulates the inclusion of vascular endothelial growth factor (VEGF) into EVs to induce tumor growth and angiogenesis but, on the other hand, myoferlin is itself included in EVs to stimulate proliferation and migration of epithelial cells that acquired myoferlin-rich EVs [104].

In a recent study, it has been reported that the protein podocalyxin is loaded into the EVs derived from *TP53* mutant PC cells. In particular, PC EVs delivered less podocalyxin protein compared to EVs from normal cells, and were able to induce increased migration of fibroblasts and extracellular matrix (ECM) reorganization. Moreover, podocalyxin levels increased after knockdown of *TP53* mutant and the effects on migration and ECM remodelling were reverted [105].

Protein cargo packaged into PC-derived EVs can contribute to the acquisition of resistance to gemcitabine treatment. Indeed, EVs derived from gemcitabine-resistant PANC-1 cells overexpress ephrin type-a receptor 2 (EphA2) compared to gemcitabine-sensitive cells and treatment of MIAPaCa-2 and BxPC-3 PC cells with EphA2-overexpressing EVs makes these cells chemoresistant. In contrast, treatment with the soluble form of EphA2 does not result in the same effect, demonstrating that chemoresistance is achieved only when EphA2 is vehiculated by EVs [106].

## 4. Extracellular Vesicles and Inflammation in Pancreatic Cancer

In the path to PC, acute and chronic pancreatitis may represent intermediate stages. Acute pancreatitis is characterized by inflammatory-driven auto-digestion of the pancreatic tissue that can evolve to edema, hemorrhage, and apoptosis or necrosis of parenchymal cells [107]. CP, instead, is a consequence of diseases characterized by pro-inflammatory pancreatic secretion and fibrosis with a higher risk of evolution to PC [108].

Due to their ability to shuttle intracellular components and biomolecules—including cytokines—among close and distant targets, EVs are now being investigated as alleged mediators in the development of pancreatic inflammation and carcinogenesis [22]. Higher concentrations of circulating EVs with a specific proteomic profile were identified in a rat model of taurocholate-induced acute pancreatitis compared with control rodents [109]. Immunofluorescence experiments on these animal models revealed that PKH26 fluorescent-labeled EVs originated mainly from the liver, which retains most of the EVs from the peritoneal cavity, and immune cells [109]. These vesicles are able to trigger a series of inflammatory reactions that ultimately lead to fibrosis and calcification of the parenchymal pancreatic tissue [109]. In vitro experiments have also shown that these vesicles can break through the lung alveolar endothelial barrier and be internalized by macrophages. Here, EVs activate resident macrophages by inducing their polarization from M2 to M1 and leading to a pro-inflammatory phenotype [109]. In this setting, multi-organ damage mediated by EVs originating in experimental acute pancreatitis and accompanied by overt inflammation may represent an EV-mediated systemic complication of pancreatitis [109]. In mouse models of acute pancreatitis, circulating EVs have also been shown to contribute to pancreatitis-associated lung injury by triggering an innate inflammatory response via the activation of the nucleotide binding oligomerization domain (NOD)-like receptor protein 3 (NLRP3) and pyroptosis of alveolar macrophages [110]. Of note, EVs derived from the plasma of rats with taurocholate-induced acute pancreatitis showed a much higher proinflammatory power on macrophages compared with those isolated from the ascitic fluid of these animals [111]. This characteristic was ascribed to EV enrichment with miRNAs having specific proinflammatory properties [111].

Along with the development of an inflammatory milieu, EVs produced by cancer cells can also mediate the transformation of immune cells towards a phenotype supporting immunosuppressive and pro-tumorigenic environment [112]. Indeed, tumor-associated antigens are shuttled within EVs produced by cancer cells that are presented via MHC to DCs for further processing and tumor-specific T lymphocytes immune response activation [113]. An escape from immune surveillance can be achieved by the inhibition of lymphocyte activation and survival [22]. DCs are the most relevant antigen presenting cells (APCs) in humans and trigger immune responses by eliciting the expression of Toll-like receptors (TLRs) and IL production. Within the TLR repertoire, TLR4 is pivotal in mediating the antitumor activity of DCs [114]. DC dysregulation has been indicated as a mechanism of immunosuppression elicited by EVs s produced by PC cells [115]. This process seems to be regulated by miR-203 carried within EVs derived from PC. Following their uptake by DCs, these vesicles can induce an increase of intracellular levels of miR-203 that inhibits the expression of TLR4, TNF-α, and IL12, eventually leading to DC dysfunction [115]. Moreover, miR-212-3p-positive PC EVs are also able to inhibit the DC role of APCs towards T lymphocytes by reducing the expression of the MHC II and associated regulatory factors [56].

EV lncRNAs can also inhibit the immune response both by binding to surface molecules and by transmitting immunosuppressive cytokines, such as TGF-β1 and IL-10 [116,117]. Chen et al. have discovered that the lncRNA ENST00000560647 is highly expressed in EVs, interacts with miRNAs that are involved in PC, and facilitates immune escape. The authors showed that treatment of DC with PDAC-derived EVs induced high increase of lncRNA ENST00000560647 compared with control DC cells [118].

EVs derived from PC cells have also been involved in the suppression of adaptive and innate antitumor responses. Indeed, the lipid surface of plasmatic EVs from PC patients has been found to be enriched with tumor-associated antigens. In particular, these vesicles display B cells targets that can induce the production of plasma autoantibodies. This prevents the recognition of tumor cells by B lymphocytes, thereby facilitating an escape of cancer from complement-mediated cytotoxicity [119]. In vivo studies have shown that EVs released from PC cells can be taken up by DCs and macrophages, limiting the activation of the IL2-mediated PI3K/Akt signaling pathway and eventually promote apoptosis [120].

The most frequent mutation of *KRAS* in PC occurs in codon G12 of exon 2 (G12D in 40% of cases and G12C in 33% of cases). These point mutations prevent conversion of GTP to GDP inducing a constitutive activation of downstream signaling pathway and, finally, promoting carcinogenesis [121]. KRAS G12D is packaged into EVs which were taken up by macrophages, stimulating Signal Transducer and Activator of Transcription 3 (STAT3)-mediated fatty acid oxidation and conferring them the M2 phenotype [122].

Of the multiple mediators involved in the inflammatory process sustaining PC growth and progression, a set of inflammatory factors has special relevance in PC pathogenesis. In particular, IL6, IL10, IL13, VEGF, and TGF-β are secreted by TME inflammatory elements and cancer cells during PC [14]. For instance, the incorporation of protein Ezrin into PC EVs is able to regulate macrophage polarization conferring them a M2 phenotype which release TGF-β1 and IL10, thereby inducing ECM remodeling, angiogenesis, and promoting metastasis [123,124]. Moreover, migration inhibitory factor (MIF)-containing EVs derived from malignant pancreatic lesions are able to induce liver metastasis when injected in mice. This process is due to enhanced TGF-β expression in Kupffer cells and secretion of fibronectin by hepatic stellate cells, which start the formation of a pre-metastatic niche in the liver where bone marrow-derived macrophages and neutrophils migrate [125]. Furthermore, as an integral part of the TME and a large producer of the ECM, cancer associated fibroblasts (CAFs) release IL6, chemokine (C-C motif) ligand 2, and C–X–C motif chemokine ligand 12 (CXCL12) that eventually support tumor growth and metastases [126,127,128]. Whether this inflammatory milieu is linked to the regulation of CAF-derived EV trafficking to reprogram energy metabolism and upregulate mitochondrial oxidation observed during PC warrants investigation [129]. Indeed, this metabolic shift is able to promote glycolysis and glutamine-dependent reductive carboxylation to fuel amino acids, fatty acids, and tricarboxylic acid cycle intermediates to nutritionally deficient PC cells and promote their survival and growth [129].

EVs derived from the PC cell lines BxPC3 carrying a homozygous deletion of the SMAD4/DPC4 gene showed downregulation of VDAC1 (Voltage Dependent Anion Channel 1) and spectrin β expression, both related to calcium balance, and upregulation of glycolytic enzymes compared with SMAD4-expressing cells. These EVs activate myeloid-derived suppressor cells, creating an immunosuppressive environment through increased calcium fluxes and stimulation of glycolysis [130]. Therefore, the absence of proteins related to the immune response in EVs and the presence of proteins that confer an anti-inflammatory phenotype to macrophages may represent two mechanisms for PC cells to successfully elude the immune system, proliferate, and initiate the metastatic cascade.

Regardless of the specific metabolic control exerted over cancer cells by inflammatory factors during PC, TNF-α, IL6, TGF-β, and IL10 are shared mediators for the initiation and progression of several cancer types [131]. High levels of IL6, a well-known cytokine exerting also a pro-tumorigenic effect [132], have been found in the serum of people with PC and have been associated with tumor stage and survival [14]. IL6 seems to act as an autocrine and paracrine signal triggering tumor cell migration and invasion, as well as EMT [126]. Similarly, TGF-β, a pleiotropic cytokine with both anti-inflammatory and immune-suppressive effects, enhances tumor invasion and metastasis via EMT in late tumor stage [133]. IL10, a powerful anti-inflammatory mediator secreted by tumor cells and almost all immune cells, possesses a dual role. Indeed, by inhibiting the signaling of the nuclear factor-κB (NF-κB) IL10 can have an anti-tumor effect, whilst by mediating immunosuppression, IL10 can allow cell maturation and differentiation and thereby promote cancer immune evasion [131,134,135]. Finally, TNF-α, a master regulator of the host immune response, may or may not convey protective effects during carcinogenesis in different cancer types depending on the type of receptors recruited (i.e., TNFR1 or TNFR2) [131,136]. Upon activation, both TNFR1 (also known as p55) which is ubiquitously expressed, and TNFR2 (also known as p75), a marker of immune cells can trigger apoptosis or NF-kB signaling. However, a concentration effect for this cytokine has been reported for the deployment of responses that can be either protective or not. Indeed, when present at low concentrations, TNF-α may sustain tumor promotion via the regulation of ROS and reactive nitrogen species (RNS) signaling, oncogene activation, and DNA damage [136]. TNF-α is produced at picogram levels by immune and pancreatic tumor cells. Moreover, the activation of this signaling pathway in PC cells has been shown to trigger the recruitment of T_reg_ cells at cancer sites and therefore increase tumor cell invasiveness in vitro and in vivo [137,138]. TNF-α levels in human PC tissues were associated with chemoresistance but not tumor stage. Moreover, higher TNF-α expression levels were correlated with shorter survival, thereby suggesting this inflammatory molecule as a prognostic marker of PC [139]. Following TNF-α treatment of a human PC cell line, increased invasiveness which correlates with stemness and increased expression of the epidermal growth factor receptor (EGFR) has been observed [140].

The strong association found between the expression of specific pattern of inflammatory molecules and PC severity (i.e., tumor size, stage, and metastasis) has led to propose some of these mediators as prognostic biomarkers for PC. However, whether a causal relationship exists between the expression of the above-mentioned cytokines and PC outcomes (e.g., severity and/or patient survival) has yet to be demonstrated.

## 5. Extracellular Vesicles and Tumor Microenvironment: From CAFs to PC

Extensive desmoplasia is a hallmark characteristic of PC [141,142]. Indeed, the majority of pancreatic tumor bulk consists of fibroblasts [141,142]. A large body of evidence suggested that among fibroblasts, CAFs contribute to the PC phenotype of chemoresistance, invasion, metastasis, and immune tolerance [143]. Furthermore, the secretion of several molecules leading to highly fibrous tumors and to tumor growth is ascribable to CAFs [144,145,146]. For this reason, a possible explanation for general PC treatment failure is the complex architecture and cellular composition of this cancer and today fibroblast-depleting therapies are emerging. Richards and coauthors (2017) have demonstrated that CAFs play an active role in the regulation of survival and cancer cell proliferation. Indeed, these CAFs are intrinsically resistant to gemcitabine and they showed significantly miR-146a higher expression compared to untreated CAFs. This miRNA is able to induce chemoresistance-inducing factor Snail and, after drug exposure, CAFs increase the release of exosomes (the endosomal origin of these vesicles was demonstrated by the authors allowing to define them as exosomes) delivering Snail into recipient cells and, thus, promote cell proliferation and gemcitabine resistance [147]. Moreover, CAFs also transfer miR106b to PC cells through EVs and promote gemcitabine resistance by targeting tumor protein P53 inducible nuclear protein 1 (TP53INP1) [148]. The importance of CAFs and their EV secretion was underlined by the possibility to use them as a target of PC therapy. To this purpose, it was demonstrated that CAF treatment with calcitriol, vitamin D active type is able to induce signaling mediated by vitamin D receptor (VDR) and consequent downregulation of miR-10a-5p in EVs from CAFs and also in EV-recipient PC cells [149]. This study provides evidence that acting on miR-10a-5p levels by VDR activation may have an antitumoral effect and may improve the therapeutic response by prompting stromal remodeling [150].

Leca and co-workers [151] have analyzed the stromal signature of CAFs and they found that annexin A6/LDL receptor-related protein 1/trombospondin 1 (ANAX6/LRP1/TSP1) complex formation is restricted to CAFs and improves PC cell survival and migration. The authors demonstrated that uptake of CAF-derived ANXA6+ EV carrying the ANAX6/LRP1/TSP1 complex determined the increase of PC aggressiveness. They observed impairment of PC and metastasis occurrence after depletion of ANAX6 and consequent reduction of ANAX6/LRP1/TSP1 complex formation in CAF. Conversely, they observed an enhance of tumorigenesis after CAF-derived ANXA6+ EV injection. Finally, it is important to underline that crosstalk between stromal and cancer cells might also have a tumor suppressor action [152]. Indeed, it was demonstrated that selective packaging of miRNAs into EVs determines an enrichment of stromal specific miR-145 in EVs secreted by tumor-associated stroma. These vesicles are able to induce apoptosis in cancer cells after stromal secretion and PC uptake suppressing tumor growth. This discovery might have future implications for therapy of unresectable PC [152].

## 6. Extracellular Vesicles in Pancreatic Cancer Diagnosis and Prognosis: Methodologies of Purification and Clinical Implications

### 6.1. Methodologies for High-Quality Purification of Extracellular Vesicles

Due to the small size, isolation of exosomal pure samples and their quantification is the major hindrance EV research. Today, ultracentrifugation, ultrafiltration, size exclusion chromatography, precipitation, immunoaffinity-based capture are the current methods employed to isolate EV fraction from biological fluids and cellular medium [153]. Isolation easier and faster with larger and purified yields of EVs has been obtained thanks to the expeditious development of these methods, albeit there are many disadvantages for each of them. For instance, the ultracentrifugation method is based on density, size, and shape-based sequential separation consisting of several centrifugation steps to remove cells, debris, and finally, pellet EVs. Characterized by reduced cost, reduced contamination risks, and large sample capacity, it yields large amounts of EVs, but this method requires high equipment costs, long run time, labor-intensive and low portability, without neglecting damage induced to EVs for high-speed centrifugation [154]. With the ultrafiltration method, EVs are concentrated on the pore-containing membrane and purified on the basis of differences between their size among other particulates. Albeit ultrafiltration is a fast and easy method, EVs are separated with moderate purity and they may be clogged and trapped to membranes [155]. The size exclusion chromatography (SEC) requires a long runtime, but it separates macromolecules on the basis of their size, applying fluid on a column packed with porous, polymeric beads with the advantage of allowing precise separation of both large and small EV without affecting EV structure [156]. By using water-excluding polymers, the precipitation method is able to purify EVs altering their solubility or dispersibility. This procedure is very easy to use and induce only a mild effect on isolated EVs, but it has the risks to co-precipitate also non- EV contaminants, such as proteins or polymeric materials [157] The immunoaffinity-based capture is based on the specific interaction between membrane-bound antigen of EVs and immobilized antibody allowing to obtain high purified EV fraction with a high possibility of subtyping. Nevertheless, the cost of the reagents is very high and it is essential to know EVs tags. Moreover capacity and yields are low and isolated EVs may loose their functional capacity [158]. Finally, the purification based on microfluidics technologies, in which channels with micrometric dimension are used to manipulate small amounts of fluids using capillary forces, has the advantage of requesting small volume sample and of being low cost, but also the sensibility of determination is very low. The described limitations of the enunciated methods highlight the urgent need for developing novel approaches to rapidly, simply, and cost-efficiently isolate EVs. The achievement of this objective would greatly advance research into EV and their content role as disease biomarkers and as therapeutic systems.

### 6.2. Extracellular Vesicles as Diagnostic and Prognostic Biomarkers of Pancreatic Cancer

A lot of cancer biomarkers are commonly used in clinical practice for diagnostic or prognostic purposes by simply analyzing their presence in patients’ biological fluids. An example is represented by serum carbohydrate antigen (CA19-9), which is today the only FDA-approved biomarker for PC diagnosis [159]. Nevertheless, the limits of this diagnostic approach are low sensitivity, high rate of false-positive for patients with benign diseases and the degradation reaction to which these markers are exposed in the bloodstream, therefore the diagnosis comes in an advanced stage for many patients [160,161]. In this scenario, EVs could represent a more useful diagnostic and prognostic tool as they are abundant and highly stable in biological fluids and, in particular, in the blood; EVs can be isolated from small volumes of serum or plasma and the analysis of their content could be used in the diagnosis and prognostication of PC [162].

Interestingly, a study based on the analysis of five pancreatic cancer-initiating cell markers (CD104, MET, EpCAM, Tspan8, and CD44v6) and four miRNAs (miR-4306, miR-3976, miR-4644, and miR-1246) in serum EVs demonstrated that evaluation of these markers allowed to distinguish patients with PC from controls, patients with chronic pancreatitis (CP), and patients with benign pancreatic lesions, with 93% specificity [57].

Moreover, Joshi and co-workers have developed a system based on glass substrate-bound gold nanoprisms functionalized with complementary oligonucleotides to detect miR-10b in EVs of PC patients, isolated from plasma by ultracentrifugation. This assay detects very small increases in miR-10b abundance and therefore it could be used for early-stage PC detection and for monitoring PC recurrence [163]. The diagnostic value of miR-10b for PC has been confirmed by observing its overexpression in EVs isolated from patients’ blood by ultracentrifugation. Furthermore, the increased plasma levels of EV miR-10b, along with miR-196a and miR-1246, in PC patients compared with CP or healthy controls highlights the potential role of EV miRNAs as biomarkers for PC diagnosis [163,164].

A further study analyzed the plasma of patients with CP and PC compared with healthy controls and found that miR-21, miR-155, miR-31, miR-let-7a, miR-221, miR-181a, miR-935, and miR-508 were differentially expressed [162]. Another study showed that PC samples may be discriminated from healthy controls and CP for their high levels of EV miR-21, miR-10b, miR-30c, and miR-181a, together with low levels of miR-let7a [165]. Recently, it was also described that miR-17-5p is overexpressed in PC-circulating EVs with specificity and sensitivity of 92.6% and 72.7%, respectively. This overexpression is enhanced in the metastatic stage suggesting that miR-17-5p could be a possible biomarker for PC detection and staging [166]. Furthermore, it was shown that miR-3940-5p/miR-8069 ratio was elevated and specific in PC patients and, in particular, in urine EVs compared with sera EVs, representing a potent diagnostic tool for PC [167].

One of the proteins abundant in EVs derived from PC cells is glypican-1 (GPC1), a cell surface proteoglycan, useful for the early detection of PC. Indeed, circulating EVs of patients with PC show significantly increased levels of GPC1 compared to healthy subjects and to patients with benign pancreatic diseases, with 100% of specificity and sensitivity [168]. Moreover, Costa-Silva and co-workers isolated EVs from patients’ plasma by ultracentrifugation and the following ELISA assay showed that PC-derived EVs of stage I patients that later developed liver metastasis had higher expression of MIF, compared to EVs of patients with non-progressing-PC. These data suggest that EV MIF could be a prognostic marker for PC liver metastasis [125]. Also, circulating PC-EVs contain high levels of EphA2 compared to healthy controls. This increased expression correlates with shorter overall survival and a multivariate analysis showed that it is a negative prognostic factor. Furthermore, high EphA2 expression was associated with shorter recurrence-free survival, suggesting that this EV protein could represent a biomarker for a poor prognosis in PC patients [169].

Interestingly, it has been demonstrated that formation of metastasis in the liver or in the lungs is influenced by the integrin content in EVs derived from PC cells. Indeed, integrin αvβ5-positive EVs usually give rise to liver metastasis, while integrin α6β4- and α6β1-positive EVs indicate that lungs will be more likely affected. This different expression pattern of integrins could be useful as a prognostic marker to predict the site of metastasis [170]. Moreover, the presence of ANXA6^+^ into CAF-derived EVs was described only in PC patients’ sera compared to healthy control, representing a potential marker of PC grade [151].

As with EV miRNAs and proteins—lncRNAs also, due to their high tissue specificity—could potentially be included in the plethora of PC biomarkers to identify and distinguish between different cancer subtypes [171,172].

A recent study has demonstrated that the origin of the tissue could be deduced by analysing plasmatic EVs lncRNA allowing to distinguish a patient’s pancreatic cancerous profile from that of an healthy control. In the same study, it was reported that eight EV lncRNAs (i.e., FGA, KRT19, HIST1H2BK, ITIH2, MARCH2, CLDN1, MAL2, and TIMP1) represent a collection of a diagnostic signature that allows distinction between patients with stage 1 PDAC and those with stage III tumors [173]. In addition, a recent study demonstrated that lcn-SOX2OT is upregulated in PC EVs derived from patients’ plasma and its expression correlated with TNM stage and overall survival rate of PC patients. Moreover, they found a reduction of EV lcn-SOX2OT in postoperative blood samples of PC patients suggesting that this lcnRNA could be a marker for prognosis of this neoplasia [84].

The potential prognostic and diagnostic power of circulating lncRNA HULC for PC was underlined by a study where the authors found EV-circulating HULC within PDAC patients and compared it to CA19-9 [174]. In this study, HULC was described as an ideal marker to distinguish PDAC patients from healthy individuals, similarly to CA19-9. Finally, the analysis of EV RNA cargos from blood samples of healthy controls, patients with IPMN, and patients diagnosed with PDAC has shown an increased expression of lncRNAs MALAT1 (metastasis-associated lung adenocarcinoma transcript 1) and CRNDE (Colorectal Neoplasia Differentially Expressed) in serum EVs from IPMN and PDAC compared to healthy EVs. Thus, it is reasonable to consider the diagnostic power of these lncRNAs a promising potential tools for precision medicine applications, allowing not only to distinguish PDAC patients from healthy subjects, but also to identify different stages of the disease and individual differences [174].

## 7. Conclusions

EVs play a key role in PC cancer progression. In this review, we summarized the role of these nanovesicles in the context of their miRNA, lncRNA, and protein cargos in the acquisition of tumorigenic properties that represent different hallmarks of PC cancer progression: (i) ability to proliferate; (ii) capability to induce cancer mass angiogenesis; (iii) ability to invade TME and metastasize in ectopic sites distal from the primary tumor; (iv) chemoresistance; (v) ability to elude immunosurveillance; and (vi) ability to create an inflammatory contest (Table 1). The importance in detecting and analyzing cancer-specific EV cargos in biological fluids from cancer patients has emerged in recent years and is referred to as liquid biopsy. Given the minimally invasiveness of blood analyses, the liquid biopsy allows to easily extract information from the tumor in real time and can be repeated over time [18]. Difficulties in early diagnosis and in curative surgical resection make PC one of the most aggressive and deadly tumors. For these reasons, the identification of PC diagnostic and prognostic markers is a major research focus. The characterization of EV content and function in PC will allow novel biomarkers to be identified for diagnostic and prognostication purposes, and could also guide the development of innovative therapeutic approaches.

## Figures and Tables

**Table 1:** Extracellular vesicle (EV) miRNAs, lncRNAs, and proteins in PC.

EV Content	Role in PC	Mechanism	Diagnostic/ Prognostic Marker	Ref.
miR-27a	Induce proliferation, invasion and angiogenesis	Suppression of BTG2 expression	-	[63]
miR-339-5p	Inhibit migration and invasion	Downregulation of ZNF689	-	[63]
miR-222	Induce proliferation and invasion	Downregulation, phosphorylation and nuclear exit of p27 through PPP2R2A/Akt pathway	-	[64]
miR-301a-3p	Promote invasion and metastasis	PTEN/PI3K signaling cascade	-	[66]
miR-1246	Induce resistance to gemcitabine, metastasis, invasion, cancer stemness and angiogenesis. Detected in the patient’s serum	Inhibition of CCNG2 expression	Yes	[69] [57,164]
miR-155	Induce resistance to gemcitabine	Downregulation of DCK	-	[70]
miR-210	Induce resistance to gemcitabine, invasion and metastasis	Induction of ZEB1 and ZEB2 expression	-	[73]
miR-23b-3p	Promote proliferation, migration and invasion	Correlate with diagnosis of PC and with CA19-9 levels	-	[74]
miR-196b-5p/194-5p	Promote resistance to radiation, cell survival	Regulation of E2F3 and HMGA2	-	[75]
miR-203	Induce immunosurveillance escape	Inhibition of TLR4, TNF-α and IL12 expression, DC dysfunction	-	[115]
miR-212-3p	Induce immunosurveillance escape	Inhibit the DC role of APCs towards T lymphocytes by reducing the expression of the MHC II and associated regulatory factors	-	[56]
miR-17-p and miR-21	High levels are associated to PC	Correlate with metastasis and advanced PC	Yes	[166]
miR-10b, miR-31, miR-30c, miR-181a, miR-221, miR-935, miR-508, miR-196a,	High levels in the plasma are associated to PC	Correlate with diagnosis of PC	Yes	[162,163,164,165]
miR-let7a	Low levels in the plasma are associated to PC	Correlate with diagnosis of PC	Yes	[165]
miR-451a	Associate to tumor size and stage of PC	Correlate with recurrence and survival	Yes	[175]
miR-3940-5p/miR-8069 ratio	High levels are specific in PC patient	Correlate with diagnosis of PC	Yes	[167]
miR-4306, miR-3976, miR-4644	Detection in the patient’s serum	Allow to distinguish between patients with PC, CP and benign pancreatic lesions	Yes	[57]
lncRNA SBF2-AS1	Inhibit of progression and increase of chemoresistance	Increase of miR-122-5p level	-	[81]
lncRNA NONHSAT105177	Inhibit proliferation and migration	Modulation of cholesterol pathway	-	[82]
lncRNAUCA1	Induce migration and tubulogenesis	Sponge of miR-96p	-	[83]
lncRNA SOX2OT	Promote EMT, invasion and metastasis	Associated with tumor stage	Yes	[84]
lncRNA HULC	Modulate cellular signaling, invasion and migration	Interact with TGF-β	Yes	[159]
lncRNA ENST00000560647	Induce immunosurveillance escape	Induce DC dysfunction	-	[118]
lncRNA FGA, KRT19, HIST1H2BK, ITIH2, MARCH2, CLDN1, MAL2, TIM1	Allow to distinguish between patients with stage I and stage III tumors	Diagnostic signature	Yes	[173]
lncRNA MALAT1 and CRNDE	High levels in serum of PC patient	Correlate with diagnosis of PC	Yes	[174]
GNA12/13	Contribute to tumor growth, angiogenesis, EMT, migration and invasion	Activation of Ras, Rac, rho, CDC42 pathways	-	[[91]
ZIP4	Enhance migration, invasion and chemoresistance	Not mentioned	-	[95]
AEP	Promote invasion	Activation of PI3K pathway	-	[96]
CD44v6	Promote migration and invasion	Activate Wnt/β-Catenin pathway and increase PAI-1, MMP and TIM-1	Yes	[97] [57]
Lin28B	Promote invasion and metastasis	Activation of Lin28B/let-7/HMGA2/PDGFB pathway	-	[108]
Integrin α6β4/	Induce dissemination of metastasis to liver and lung tissues	Integrin-mediated organotropic incorporation of EVs	Yes	[170]
Tspan8, CD106, Cd49d	Promote neovascularization, tumor growth, metastasis	Increase expression of chemokine and receptor such as CXCR4 and EGFR3	Yes	[100,101] [57]
Myoferlin	Induce migration, invasion, angiogenesis, proliferation	Regulation of mitochondrial organization and energy production, stimulate inclusion of VEGF into EVs	-	[102,103,104]
Podocalyxin	Induce migration and ECM organization	Correlation with TP53 mutations	-	[105]
EphA2	Resistance to gemcitabine	Not mentioned	Yes	[106]
GPC1	Associate to early stage of PC	Correlation with KRAS mutations	Yes	[168]
KRAS G12D	Correlate with poor survival	Stimulation of macrophage switch to an M2-like pro-tumor phenotype via STAT3-dependent fatty acid oxidation	-	[122]
Ezrin	ECM remodeling, angiogenesis, and metastasis	Stimulation of macrophage switch to an M2-like pro-tumor phenotype and release of TGF-β1 and IL-10	-	[123,135]
MIF	Promote the liver pre-metastatic niche formation	Up-regulation of TGF-β expression and fibronectin secretion	Yes	[125]
ANXA6	Improve PC cell survival and migration	Formation of ANAX6/LRP1/TSP1 complex	Yes	[151]

## Data Availability

Not applicable.

## Abbreviations

PC—Pancreatic Cancer; CP—Chronic Pancreatitis; EV—Extracellular vesicle; EMT—Epithelial Mesenchymal Transition; ECM—Extracellular Matrix; BTG2—B-cell translocation gene 2; ZNF689—zinc finger; PPP2R2A—Protein Phosphatase 2 Regulatory Subunit B alpha; PTEN—Phosphatase and Tensin Homolog; PI3K—Phosphatidylinositol 3 Kinase; CCNG2—clinical significance of cyclin G2; DCK—Deoxycytidine Kinase; ZEB1/2—Zinc finger E-box-binding homeobox; MALAT1—metastasis-associated lung adenocarcinoma transcript 1; CRNDE—Colorectal Neoplasia Differentially Expressed; CA19-9—carbohydrate antigen; E2F3—E2F transcription factor 3; HMGA2—high mobility group AT-hook 2; TLR4—Toll-like receptors; TNF-α/β—tumor necrosis factor α/β; IL—interleukin; DC—dendritic cell; GNA12/13—Guanine Nucleotide-Binding Protein Alpha; ZIP4—Zrt- and Irt-like protein 4; AEP—asparaginyl endopeptidase; CD44v6—CD44 variant isoform 6; Lin28B—Lin-28 homolog B; Tspan8—Tetraspanin 8; PAI-1—plasminogen activator inhibitor 1; MMP—metalloproteases TIM-1—tissue inhibitor of metalloproteases 1; PDGFB—platelet-derived growth factor subunit B; CXCR4—C-x-C motif chemokine receptor 4; EGFR3—Epithelial Growth Factor 3; VEGF —vascular endothelial growth factor; EphA2—ephrin type-a receptor 2; GPC1—glypican-1; MIF —migration inhibitory factor; STAT3—Signal Transducer and Activator of Transcription 3; ANXA6 —Annexin A6; LRP1—LDL receptor -related protein 1; TSP1—trombospondin 1.
